# Forkhead transcription factors: new considerations for alzheimer’s disease and dementia

**DOI:** 10.15761/JTS.1000146

**Published:** 2016-06-14

**Authors:** Kenneth Maiese

**Affiliations:** Cellular and Molecular Signaling, Newark, New Jersey 07101

**Keywords:** Akt, apoptosis, autophagy, cell longevity, forkhead transcription factors, FoxO, growth factors, erythropoietin, mechanistic target of rapamycin (mTOR), mitochondria, oxidative stress, programmed cell death, reactive oxygen species, SIRT1, sirtuins, stem cells, transcription factors, WISP1, Wnt signaling

## Abstract

Life expectancy of individuals in both developed and undeveloped nations continues to rise at an unprecedented rate. Coupled to this increase in longevity for individuals is the rise in the incidence of chronic neurodegenerative disorders that includes Alzheimer’s disease (AD). Currently, almost ten percent of the population over the age of 65 suffers from AD, a disorder that is presently without definitive therapy to prevent the onset or progression of cognitive loss. Yet, it is estimated that AD will continue to significantly increase throughout the world to impact millions of individuals and foster the escalation of healthcare costs. One potential target for the development of novel strategies against AD and other cognitive disorders involves the mammalian forkhead transcription factors of the O class (FoxOs). FoxOs are present in “cognitive centers” of the brain to include the hippocampus, the amygdala, and the nucleus accumbens and may be required for memory formation and consolidation. FoxOs play a critical role in determining survival of multiple cell types in the nervous system, drive pathways of apoptosis and autophagy, and control stem cell proliferation and differentiation. FoxOs also interface with multiple cellular pathways that include growth factors, Wnt signaling, Wnt1 inducible signaling pathway protein 1 (WISP1), and silent mating type information regulation 2 homolog 1 (*Saccharomyces cerevisiae*) (SIRT1) that ultimately may control FoxOs and determine the fate and function of cells in the nervous system that control memory and cognition. Future work that can further elucidate the complex relationship FoxOs hold over cell fate and cognitive function could yield exciting prospects for the treatment of a number of neurodegenerative disorders including AD.

## Assessing the role of FoxO transcription factors for Alzheimer’s disease and cognitive loss

### Increased life expectancy of the global population with increased prevalence of cognitive disorders

Life expectancy throughout the globe continues to rise in both developed nations as well as in large nations that are under development [1]. For example, in China, the number of elderly individuals will continue to grow from current levels of five percent to levels of approximately ten percent over subsequent decades [2]. Accompanied by this rise in age and lifespan of the world’s population is the increased incidence of chronic neurodegenerative disorders that includes Alzheimer’s disease (AD) [3, 4]. AD can be of familial or sporadic origin. Familial cases of AD occur as an autosomal dominant form of a mutated amyloid precursor protein (APP) gene as well as mutations in the presenilin 1 or 2 genes. Occurrence of familial cases of AD is relatively rare, usually presents prior to age 55 [5], and is present in less than 2 percent of all presentations [6]. Familial AD has been reported in approximately two hundred families throughout the world. Variable single-gene mutations on chromosome 1 lead to altered presenilin 2, mutations on chromosome 14 result in altered presenilin 1, and mutations on chromosome 21 lead to altered APP. For sporadic AD, almost ten percent of the population over the age of 65 suffers from this disorder [7].

### Targeting novel therapies for Alzheimer’s disease with forkhead transcription factors

With the advancing age of the global population and the progressive increase observed in life span, it is estimated that the incidence of sporadic AD will significantly increase throughout the world [7–9]. In addition, healthcare resources will be greatly impacted. In the Unites States (US) alone, greater than five million individuals are diagnosed with sporadic AD and approximately four million are under treatment at an annual cost of 3.8 billion US dollars. The onset and progression of AD is multifactorial. Underlying mechanisms that may lead to cognitive impairment involve cellular injury from β-amyloid (Aβ), tau, excitotoxicity, mitochondrial damage, acetylcholine loss, astrocytic cell injury, oxidative stress, and cellular metabolic dysfunction [10–20]. In light of the multiple pathways that may be responsible for the onset of AD, developing therapies are designed to focus on a variety of targets that include DNA methylation, deployment of monoclonal antibodies against Aβ, prevention of Aβ and tau aggregation, increased cytokine and growth factor signal transduction, mammalian target of rapamycin (mTOR) modulation, and the application of metal chelators [14, 15, 17, 18, 21–27]. However, included in this growing arsenal of potential targets are mammalian forkhead transcription factors of the O class. FoxOs may represent one of the most exciting and novel strategies for the development of therapies against AD.

### The FoxO family of proteins and modulation of FoxO transcription factor activity

More than one hundred forkhead genes and 19 human subgroups that range from *FOXA* to *FOXS* are now known to exist since the discovery of the *Drosophila melanogaster gene forkhead* [28]. Mammalian FOXO proteins that are of the O class of the forkhead box class transcription factors have the members FOXO1, FOXO3, FOXO4, and FOXO6 [29]. In relation to the nomenclature for forkhead box class transcription factors, an Arabic number is used with the designation of "Fox", followed by a subclass or subgroup letter, and then the member number is listed within the subclasses of the Fox proteins [30]. For human Fox proteins, all letters are capitalized. Only the initial letter is listed as uppercase for the mouse and for all other chordates the initial and subclass letters are in uppercase [31–34]. FoxO proteins are transcription factors and bind to deoxyribonucleic acid [DNA] through the FoxO-recognized element in the C-terminal basic region of the forkhead DNA binding domain [35, 36]. It is important to note that multiple mechanisms may affect forkhead protein binding to DNA. These mechanisms can involve variations in the N-terminal region of the recognition helix, changes in electrostatic distribution, and blockade of nuclear translocation of FoxO proteins [37–40]. Following forkhead binding to DNA, target gene expression is repressed or activated through fourteen protein-DNA contacts with the primary recognition site located at α-helix H3 [41]. In addition, phosphorylation or acetylation of forkhead proteins can block FoxO activity and alter the binding of the C-terminal basic region to DNA to prevent transcriptional activity [42].

Phosphorylation of forkhead transcription factors can be controlled by the serine-threonine kinase protein kinase B (Akt) [7, 43–48]. Akt phosphorylates FoxO proteins that result in the binding of FoxOs to cytoplasmic 14-3-3 proteins. This action prevents nuclear translocation of FoxOs and blocks the transcription of target genes that promote apoptosis [49–52]. Other pathways in addition to Akt also can lead to the phosphorylation and inactivation of FoxO proteins. The serum- and glucocorticoid-inducible protein kinase (SgK), a member of a family of kinases termed AGC (protein kinase A/protein kinase G/protein kinase C) kinases that includes Akt, phosphorylates FoxO3a to sequester forkhead proteins in the cytoplasm [53]. Since Akt and SgK phosphorylate FoxO proteins at different sites, it may be possible to exploit this knowledge to allow for increased options for controlling forkhead protein activity. Yet, some protein kinases such as mammalian sterile 20-like kinase-1 (MST1) can phosphorylate FOXO proteins and disrupt the binding to 14-3-3 which then allows FOXO nuclear translocation and subsequent death in neurons [38], further suggesting that the phosphorylation site of FoxO proteins is crucial in determining the activity of forkhead transcription factors.

Ubiquitylation and acetylation also control post-translational modification and activity of FoxO proteins [54, 55]. For example, Akt also leads to the ubiquitination and degradation of FoxO proteins through the 26S proteasome [55, 56]. Agents that can prevent the ubiquitination and degradation of FoxO proteins may serve as important entities to induce apoptotic cell death in cancers that can be tied to silent mating type information regulation 2 homolog 1 (*Saccharomyces cerevisiae*) (SIRT1) [57, 58]. In a similar vein, SIRT1 activity also can lead to enhanced cell survival such as in the nervous system through modulation of FoxO activity [59–63].

FoxO proteins are acetylated by histone acetyltransferases that include p300, the CREB-binding protein (CBP), and the CBP-associated factor. Once acetylated, FoxO proteins translocate to the cell nucleus but have diminished activity since acetylation of lysine residues on FoxO proteins has been shown to limit the ability of FoxO proteins to bind to DNA [64]. Furthermore, acetylation can increase phosphorylation of FoxO proteins through Akt [64]. In contrast, increased activity of FoxO proteins such as during deacetylation can lead to cytochrome c release and caspase-induced apoptotic death [37, 48, 63, 65–68].

### FoxO proteins drive cellular survival through apoptosis and autophagy

FoxO proteins may play multiple roles in the nervous system [36, 69]. For example, forkhead transcription factors, such as FoxO3, may control cerebral endothelial vascular cell survival [70, 71], oxidative stress injury in mouse cerebellar granule neurons [72], neonatal hypoxic-ischemic encephalopathy [73], erythroid cell growth [74], and hippocampal neuronal injury [51, 75]. FoxO transcription factors also appear to be involved in memory formation and consolidation [76] especially since these transcription factors are present in several regions of the brain, such as the hippocampus, the amygdala, and the nucleus accumbens [77, 78].

FoxO proteins oversee cell survival in the nervous system through the programmed cell death pathways of apoptosis and autophagy [79, 80]. Under a number of conditions, FoxO transcription factors lead to apoptotic cell death that can involve oxidative stress [81]. In regards to FoxO1 or FoxO3a, inhibition or gene knockdown of these transcription factors leads to stroke reduction by estradiol [52], protects against microglial cell demise during oxidative stress [82] and β-amyloid (Aβ) exposure [83], promotes the protective effects of metabotropic glutamate receptors [65], increases neuronal cell survival through nicotinamide adenine dinucleotide (NAD^+^) precursors [66], and provides trophic factor protection with erythropoietin (EPO) [37, 50, 70, 74] and neurotrophins [84–86]. Other pathways also rely upon the down-regulation of FoxO to foster cellular survival and block apoptosis. Wnt signaling pathways [87] with Wnt1 in microglial cells of the central nervous system prevents apoptosis through the post-translational phosphorylation and sequestration of FoxO3a in the cytoplasm to prevent the loss of mitochondrial membrane permeability, cytochrome c release, Bad phosphorylation, and activation of caspases [68]. Wnt1 inducible signaling pathway protein 1 (WISP1), a target of Wnt signaling [88, 89], also protects neurons through the post-translational phosphorylation of FoxO3a, sequestration of FoxO3a in the cytoplasm with protein 14-3-3, and limiting the deacytelation of FoxO3a [51]. Neuroprotective trophic factors and cytokines, such as EPO [30, 37, 70], also use Wnt signaling to offer cellular protection through the inhibition of FoxO proteins. However, other studies show that in some cellular populations, such as mouse hematopoietic stem cells, the conditional deletion of FoxO1, FoxO3a, and FoxO4 can lead to an increase in reactive oxygen species [ROS] [90], suggesting that FoxO proteins in some environments can be beneficial in the regulation of oxidative stress [91, 92].

During the induction of autophagy [93, 94], FoxO proteins may enhance cellular survival. In experimental models of full-length mutant Huntingtin (mHtt) transgenic mice, ectopic expression of FoxO1 enhances autophagy and toxic mHtt protein clearance in neuronal cell cultures [95]. Activation of FoxO proteins and autophagy also may prevent apoptotic cell injury during oxidative stress such as in chondrocytes [96]. Loss of FoxO activity with reduction of autophagy during aging may contribute to neuronal dysfunction and the induction of Aβ production [97]. However, during development, repression of FoxO activity and blockade of autophagy may be necessary for improved cell survival. For example, loss of Foxo that prevents autophagy induction and the combined inhibition of reaper pro-apoptotic genes leads to long-term survival of neuroblasts and neurogenesis in centers responsible for learning and memory in Drosophila [98].

### FoxO proteins oversee stem cell survival development and differentiated cell survival

FoxO proteins can influence neuronal precursor development and the maintenance of neurons [3, 99]. Loss of Foxa1 and Foxa2 in mice results in reduced striatal dopamine metabolites, loss of dopaminergic cells, and progressive locomotor deficits [100]. In signaling pathways that involve WISP1, FoxO may be detrimental to stem cell development. WISP1 expression is up-regulated during stem cell migration [101] and affects induced pluripotent stem cell reprogramming [102, 103]. Since WISP1 requires β-catenin for the differentiation of marrow derived mesenchymal stem cells [104], FoxO may interfere with this process and bind to β-catenin that ultimately blocks stem cell development [88, 105]. In addition, the growth factor EPO [106] requires control of FoxO3a activity to promote eythroid progenitor cell development [74, 107–109]. Glial cell line-derived neurotrophic factor also inhibits FoxO1 and FoxO3a activity to promote rat enteric nervous system precursor development [110].

In differentiated cells of the nervous system, FoxO activation may impair cellular survival [111]. Toxin exposure in cortical neurons that activates FoxO3a and p27 (kip1) transcription leads to apoptosis [112]. In addition, FoxO3a association with cell cycle induction proteins has been suggested to result in neuronal apoptotic cell death [72]. Manganese toxicity, a potential factor in neurodegenerative disorders such as Parkinson’s disease [113], has been tied to astrocyte cell death through increased expression and activation of FoxO proteins [114]. Protection of cells in the nervous system occurs with the inhibition of FoxO activity and the blockade of FoxO translocation to the nucleus [115]. Furthermore, FoxO3 inactivation is necessary during antioxidant administration for the protection of cortical neurons and hippocampal neuronal cell lines in the presence of excitotoxicity [116] and in experimental models of AD with Aβ toxicity [75]. Protection of primary hippocampal neurons by group I metabotropic receptors during oxidative stress also requires the phosphorylation and inactivation of FoxO3a and the blockade of caspase activation by FoxO3a [65]. EPO has been shown to offer neuronal and vascular cell protection [117, 118] through pathways that inactivate FoxO proteins, such as FoxO3a [50, 74]. Knockdown of FoxO3a and prevention of nuclear shuttling leads to the increased survival in microglial cells and neurons of the nervous system [51, 68]. Cortical neurons [119] and vascular cells [37, 70, 120, 121] also are protected through inhibitory phosphorylation of FoxO3a and the nuclear export of this protein during periods of elevated glucose.

On the flip-side, FoxO protein activity is sometimes necessary for neuronal protection. FoxO proteins such as FoxO3 may be important for the control of autophagic flux in Parkinson’s disease [122]. A small degree of FoxO3 activity blocks nigral neuron cell death by reducing α-synuclein levels and fostering the accumulation of autophagic vacuoles containing lipofuscin [122]. FoxO3a may be necessary for cochlear auditory activity and the maintenance of synaptic function [123]. Increased FoxO protein expression prevents neurodegenerative disease and adverse behavioral deficits during selenium exposure that may linked to the development of amyotrophic lateral sclerosis [124]. Activation of FoxO proteins also may be protective during aging such that loss of FoxO3a activity results in decreased manganese-superoxide dismutase and enhanced cell injury with aging [125]. Loss of FoxO results in decreased survival and locomotive activity in Drosophila models of Aβ toxicity [126]. Yet, it should be noted that the level of FoxO activity as well as associated pathways that involve SIRT1 may be critical for survival and function of cells in the nervous system. If one considers other systems of the body, it has been suggested that a controlled up-regulation of FoxO3a and SIRT1 expression in cardiac tissue may be beneficial during exercise [127]. Levels of SIRT1 that are less than 7.5-fold are associated with catalase expression that is also controlled by FoxO1a to possibly reduce cell injury during oxidative stress. In contrast, elevated levels of SIRT1 at 12.5-fold can result in cardiomyocyte apoptosis and decreased cardiac function [128].

### Determining the role of FoxO proteins in alzheimer’s disease and cognitive impairment

FoxO proteins are deacetylated by histone deacetylases, such as SIRT1 [56, 129–131], that can be beneficial under certain conditions. SIRT1-mediated deacetylation of FoxO1 leads to starvation-induced increases in autophagic flux that can maintain cardiac left ventricular function during periods of starvation [132]. SIRT1 can promote cortical bone formation with osteoblast progenitors by deacetylation of FoxOs and preventing FoxO protein from inhibiting Wnt signaling through the binding of FoxO to β-catenin [133]. In addition, it is important to note that SIRT1 also can modulate activity of FoxOs under other conditions to increase cell survival in the brain [15] through the inhibition of FoxOs [51, 70, 121, 134].

Sirtuins and FoxO proteins also may function synergistically to promote neuronal cell survival [3, 61]. In experimental cell culture models, FoxO proteins in conjunction with SIRT1 pathways may offer protection against Aβ toxicity [135]. Forkhead transcription factors, such as FoxO3a, may be dependent upon SIRT1 to reduce oxidative stress and cell injury during exposure to Aβ [136]. SIRT1-mediated deacetylation of FoxO1 can protect cells from ischemic injury [137]. In microglia, overexpression of SIRT3 has been linked to antioxidant expression through enhanced activity of FoxO3a [138]. FoxO proteins also can regulate SIRT1 transcription and increase SIRT1 expression [139]. Loss of FoxO and SIRT1 activity with a reduction in autophagy activity, at least in models of Drosophila, may lead to increased neuronal induction of Aβ [97].

In other studies, FoxO proteins may assist with cell injury during Alzheimer’s disease. Nuclear translocation of FoxO3 is associated with DNA damage [140] and Aβ toxicity that leads to dephosphorylation and mitochondrial translocation of FoxO3a with subsequent mitochondrial dysfunction [141], suggesting that down-regulation of FoxO3a could block Aβ toxicity. In addition, astrocyte cell death during Aβ exposure has been associated with activation of FoxO3a and the pro-apoptotic target Bim and caspase 3 [142]. Histone deacetylase 2 [HDAC2] has been shown to form a physical complex with FoxO3a that plays a role with oxidative stress-induced cerebellar granule neuron apoptosis [72]. Inhibition of forkhead transcription factor activity protects against oxidative stress and A toxicity [83, 126] that may point to new therapeutic targets for Alzheimer’s disease [7]. Furthermore, blockade of a calcineurin/FoxO3 interaction in astrocytes during Aβ exposure may decrease pro-inflammatory cytokines and protect neurons [143].

## Future perspectives

Mammalian forkhead transcription factors of the FoxO family offer exciting prospects for the treatment of cognitive neurodegenerative disorders and AD. FoxOs are present in multiple regions of the brain that can influence cognition and memory to include the hippocampus, the amygdala, and the nucleus accumbens. In addition, FoxOs may be necessary for memory formation and consolidation. In regards to cell survival, FoxOs control both apoptotic and autophagic pathways. For the most part, limiting FoxO activity is necessary to block apoptotic cell death. However, the degree that specific FoxO protein activity is reduced appears to be critical, since under some circumstances FoxO activity is necessary to protect against oxidative stress. In addition, fostering the induction of autophagy by FoxOs is required for conditions that involve neurogenesis and memory development. FoxOs are intricately involved with multiple pathways that include growth factors, such as EPO and neurotrophins, Wnt signaling, WISP1, and SIRT1. With each of these pathways, post-translational modification of FoxO proteins and subsequent FoxO cellular activity can influence how FoxOs drive cellular survival and potentially affect cognitive function. Future work that continues to tease apart the complex relationship that FoxOs hold for cognitive function should bear significant fruits for the development of new strategies to treat neurodegenerative disorders such as AD.
